# Bioactive Polymeric Nanoparticles for Periodontal Therapy

**DOI:** 10.1371/journal.pone.0166217

**Published:** 2016-11-07

**Authors:** Raquel Osorio, Camilo Andrés Alfonso-Rodríguez, Antonio L. Medina-Castillo, Miguel Alaminos, Manuel Toledano

**Affiliations:** 1 Dental School. University of Granada. Colegio Máximo, Campus de Cartuja s/n. 18017 Granada, Spain; 2 Tissue Engineering Group, Department of Histology, University of Granada, 18012, Granada, Spain; 3 NanoMyP. Spin-Off Enterprise from University of Granada. Edificio BIC-Granada. Av. Innovación 1. 18016 Armilla, Granada, Spain; Institute of Materials Science, GERMANY

## Abstract

**Aims:**

to design calcium and zinc-loaded bioactive and cytocompatible nanoparticles for the treatment of periodontal disease.

**Methods:**

PolymP-*n*Active nanoparticles were zinc or calcium loaded. Biomimetic calcium phosphate precipitation on polymeric particles was assessed after 7 days immersion in simulated body fluid, by scanning electron microscopy attached to an energy dispersive analysis system. Amorphous mineral deposition was probed by X-ray diffraction. Cell viability analysis was performed using oral mucosa fibroblasts by: 1) quantifying the liberated deoxyribonucleic acid from dead cells, 2) detecting the amount of lactate dehydrogenase enzyme released by cells with damaged membranes, and 3) by examining the cytoplasmic esterase function and cell membranes integrity with a fluorescence-based method using the Live/Dead commercial kit. Data were analyzed by Kruskal-Wallis and Mann-Whitney tests.

**Results:**

Precipitation of calcium and phosphate on the nanoparticles surfaces was observed in calcium-loaded nanoparticles. Non-loaded nanoparticles were found to be non-toxic in all the assays, calcium and zinc-loaded particles presented a dose dependent but very low cytotoxic effect.

**Conclusions:**

The ability of calcium-loaded nanoparticles to promote precipitation of calcium phosphate deposits, together with their observed non-toxicity may offer new strategies for periodontal disease treatment.

## Introduction

Periodontitis is a multifactorial but an infectious disease. It is initiated by a bacterial biofilm that may provoke an immune and inflammatory response in the adjacent tissues [1]. Matrix metalloproteinases (MMPs) are enzymes present at the periodontal tissue and are produced by immunitary cells and bacteria. MMPs are proteinases able to degrade collagen leading to the destruction of the periodontal ligament and surrounding tissues [2]. To this date, there is not a therapy that guarantee to obtain a favorable outcome over stability of periodontal repaired tissue. Regenerating bone in periodontal defects around teeth or in extraction sockets, has lower clinical outcome predictability than other bone regeneration applications [3].

The use of bone graft biomaterials has demonstrated some clinical success in the treatment of periodontal defects, ridge augmentations in implant therapy or even when treating peri-implantitis [4]. Most of the employed bone graft substitutes (hydroxyapatite -HAp- and other calcium phosphates) show a relatively fast rate of biodegradation. It should be taken into account that after periodontal regeneration procedures, it might take from 6 to 12 months for occlusal reconstitution. The healing period of the alveolar bone after extraction usually needs 6 to 12 months before placement of an endo-osseous implant. Therefore, currently employed reabsorbable materials in periodontal therapy (e.g. nano/micro-sized calcium phosphate, hydroxyapatite or beta-tricalcium phosphate) may be disadvantageous, as dissolution behaviors are not always as long-lasting as required, and degradation products may not be completely cytocompatible [5,6]. The non-controllable calcium release of these materials may lead to inhibition, stimulation or no effect on osteoconductivity, osteoclastic activity or even fibrous formation at the implanted tissue. The attained effect will depend on the variations of extracellular calcium concentration values [5,6], and it is not predictable with reabsorbable materials.

The design and development of an efficient and biocompatible, non-reabsorbable material able to lead to periodontal regeneration is desirable. There is a significant scope in the application of nanomaterials to positively interfere in periodontal disease development and its progression [3,7,8]. A novel type of polymeric nanoparticles (NPs) composed by 2-hydroxyethyl methacrylate, ethylene glycol dimethacrylate and methacrylic acid connected covalently, have been produced. Nanoparticles were synthesized by polymerization precipitation (surfactant free) in a non-solvent medium, and characterized [9]. The hydrodynamic radius of nanoparticles and zeta potential were respectively 120 ± 15 nm with a polydispersity index (PDI) of 0.02 ± 0.0015, and potential zeta was -41 ± 5 mV measured in water at pH = 7 by dynamic light scattering [10]. The synthesis of particles by polymerization precipitation technique, produces non-porous particles, as a non-solvent medium is used. Sequences of anionic carboxylate (*i*.*e*. COO^-^) are along the backbone of the present polymeric NPs, and these functional groups facilitate the possibility of calcium and zinc quelation. Calcium ions have been previously employed to increase extracellular calcium concentration, because it is a potent chemical signal to produce cell migration, cell growth and for the initiation of bone remodeling [5]. Improved osteogenic ability [11] and increased osteoconductivity [12] have been associated with the use of calcium containing materials. The replacement of antibiotics by alternative antibacterial agents, such as metal oxides, may help to overcome chronic infections. Calcium and zinc nanoparticles have also been demonstrated to have significant antimicrobial activities [13,14]. Zinc may also play a role in collagen protection from MMPs degradation [15].

Polymethylmethacrylates are non-reabsorbable polymers, which have been widely used during decades to clinically fix prostheses to bone or for vertebroplasty, due to its excellent tissue compatibility [8]. However, cell viability in the presence of NPs should be ascertained.

NPs exerting biomimetic mineralization may be desirable. After CaCl_2_ immersion, calcium complexation may occur at the external surface of NPs, containing carboxyl groups, which are negatively charged. And it is hypothesized, that PO_4_^3-^ ions may ionically bond to calcium at the NPs surface. Materials containing calcium and phosphate ions will improve cementum repair and cells migration and growth [5,16]. These materials should ideally be non-cytotoxic and nanosized, to permit the particles to pass through the tissue. Moreover, nano-sized HAp has been shown to have favorable clinical outcomes for the management of intrabony periodontal defects [17,18]. Due to size effects and surface phenomena at the nanoscale, nanosized biomaterials possess unique properties over its bulk-phase counterpart. The high surface-to-volume ratio, reactivities, and biomimetic morphologies should make nano-HAp more favorable in applications for bone tissue engineering [19]. Therefore, combining the different scaffold fabrication methods with nanotechnology can provide new and improved biomaterials. It has also been reported that nano-HAp selectively increased the expression of BMP-2 in dose and time-dependent manners at mRNA and protein levels, in human periodontal ligament cells [20]. Furthermore, concentrations of Ca^2+^ and PO_4_^2-^ were not changed in cell culture supernatants, suggesting that nano-HAp functioned as a nanoparticle rather than as a possible extracellular source of Ca^2+^ and/or PO_4_^2-^ [20]. This novel mechanism explaining the action of nano-HAp encouraged the development of new strategies for periodontal intrabony defects treatment using nanoparticles [5,18,20].

The aim of the present study was to produce calcium and zinc-loaded bioactive and cytocompatible nanoparticles for periodontal regeneration therapies. The null hypotheses to be tested are: 1) controlled calcium and zinc quelation on polymeric NPs is not possible, 2) biomimetic precipitation of calcium phosphate (Ca/P) deposits on zinc and calcium-loaded polymeric spheres by immersion in simulated body fluid solution (SBFS) will not occur; and 3) calcium and zinc-loaded nanospheres do not permit human fibroblasts viability.

## Materials and Methods

PolymP-*n* Active nanoparticles (NPs) were acquired from NanoMyP (Granada, Spain). Particles are fabricated through polymerization precipitation. NPs are composed by 2-hydroxyethyl methacrylate (backbone monomer), ethylene glycol dimethacrylate (cross-linker) and methacrylic acid (functional monomer), as described in detail previously [8,9,21]

### Zinc and calcium complexation

The calcium and zinc chelating ability of the NPs was analyzed at two different pHs (6.5 and 8.5). 30 mg of NPs were incubated at room temperature, during 3 days under continuous shaking in 15 ml of different aqueous solutions of ZnCl_2_ or CaCl_2_ (containing zinc or calcium at 1,10,40 and 90 ppm), in order to reach the adsorption equilibrium of metal ions. Then, the suspensions were centrifuged and the particles were separated from the supernatant. The calcium and zinc chelating ability of nanoparticles (μgZn^+2^ or Ca^+2^/μg NPs) was calculated by the difference between the initial concentration of calcium and zinc, and the concentration found in the supernatants, through an inductively coupled plasma (ICP) optical emission spectrometer (ICP-OES Optima 8300, Perkin-Elmer, MA, USA) [22]. All tests were performed in triplicate. The Kruskal-Wallis and the Mann-Whitney tests were used. Statistical significance was set at p<0.05. The most effective conditions (pH:6.5; zinc or calcium at 40 ppm) to produce calcium and zinc complexation on nanoparticles surfaces were selected for Ca-NPs and Zn-NPs preparation.

### Transmission electron microscopy (TEM) characterization

Nanospheres were examined by (LIBRA 120 PLUS, Carl Zeiss SMT) and an attached energy dispersive analysis system (EDX) (Inca 300 and 350, Oxford Instruments, Oxford, UK) was used to detect effective zinc and calcium quelation.

### Acellular static in vitro bioactivity test

NPs and NPs loaded with zinc or calcium were soaked in 20 ml of SBFS [pH 7.45] in sterile flasks for 7 days [23,24]. Reagents per 1000 ml of SBFS: 8.035 g of NaCl, 0.355 g of NaHCO_3_, 0.225 g of KCl, 0.231 g of K_2_HPO_4_·3H_2_O, 0.311 g of MgCl_2_·6H_2_O, 39 g of 1M HCl,0.292 g of CaCl_2_, 0.072 g of Na_2_SO_4_, 118 g of Tris, 0 to 5 ml of 1M HCl for final pH adjustment [23,24]. After drying, in a vacuum heater during 24 h, polymeric spheres were analyzed by field emission scanning electron microscopy (FESEM) (GEMINI, Carl Zeiss SMT, Germany) at 2.5 to 3 Kv, 3.6 mm working distance, microscope was attached to an energy dispersive analysis system (EDX) (Inca 300 and 350, Oxford Instruments, Oxford, UK). Amorphous mineral deposition was probed by X-ray diffraction analysis (Bruker D8 Advance; XRD Bruker Corporation, Wien, Austria). Experimental conditions were CuKα radiation (λ = 1,5406 Å) in θ−θ scan, in a range 2 Theta from 5° to 90°, as described in detail previously [21]. All tests were performed in triplicate.

### Establishment of primary cultures of oral mucosa fibroblasts

Ten normal human oral mucosa biopsies with an average volume of 8 mm^3^ were obtained from healthy donors at the School of Dental Sciences of the University of Granada. Written informed consent was always obtained and the research protocol was approved by the Institutional Review Board (UGR) (#2014/891). To obtain primary cultures of human oral mucosa fibroblasts, tissues were enzymatically de-epithelized and the lamina propria was digested in a mixture of Dulbecco’s Modified Eagle’s Medium (DMEM) and 2 mg/mL *Clostridium histolyticum* collagenase I (Gibco BRL Life Technologies, Karlsruhe, Germany). Detached fibroblasts were collected by centrifugation and expanded in culture flasks containing DMEM supplemented with 10% fetal calf serum (FCS) and 1% antibiotic-antimycotics solution (final concentration 100 U/mL penicillin G, 0.10 mg/mL streptomycin and 0.25 μg/mL amphotericin B) (all from Sigma-Aldrich, Steinheim, Germany). Cells were incubated at 37°C in 5% carbon dioxide under standard culture conditions. The medium was changed every 3 days, and the cells were subcultured in a solution of 0.5 g/L trypsin and 0.2 g/L EDTA at 37°C for 10 min. For all experiments, cells from the first three passages of these human oral mucosa fibroblast cell cultures were used.

### Cell viability analysis

Particles were supplied as an aqueous suspension (30 mg/ml). Water was replaced with 70% ethanol and NPs were left in this solution during 12 h for sterilization purposes. Finally, the ethanol was removed, and the nanoparticles were suspended in DMEM. Fourteen study groups were analyzed in this work: 1, 10, 50 and 100% dilutions of NPs, Zn-NPs and Ca-NPs, positive controls -cells cultured in DMEM without particles- and negative controls -cells treated with 2% triton X-100 (Sigma, St. Louis, MO)-. Triton X-100 is a chemical compound that has been previously described to produce high cytotoxic effects [25]. In all cases, cells were analyzed after 24 h of exposition of the cells to each culture condition. Cell viability was evaluated by three different techniques: 1) Cell death, as determined by nuclear membrane integrity, was assessed by quantifying the liberated deoxyribonucleic acid (DNA) to the culture media. Supernatants of each sample were obtained, and 10-μL aliquots were diluted ten times in distilled water free of nuclease (Ambion-Life Technologies, Austin, TX, USA). The DNA in the medium was quantified spectrophotometrically (SmartSpec Plus, Bio-Rad, Hercules, CA, USA) at wavelengths in the range of 260–280 nm. The mean values and standard deviations of three independent experiments are reported here for each experimental group. 2) The LDH assay (Roche, Germany) detects the amount of lactate dehydrogenase (LDH) enzyme released by cells with damaged membranes as indicator of cell death. Cells were incubated with previously prepared particles dilutions, and LDH was quantified in the supernatant corresponding to each experimental group. In order to investigate particle interferences with the measurement, cell supernatants were transferred after centrifugation (10 min, 200g) in a new 96-well plate, and LDH assay was performed. For each experimental group, five independent determinations were taken. 3) By examining the cytoplasmic esterase function and cell membrane integrity with a fluorescence-based method using the Live/Dead commercial kit (Life Technologies, Carlsbad, CA, USA). This method uses calcein-AM, which is metabolically modified by living cells to a green pigment, and ethidium homodimer-1, which stains the nuclei of dead cells in red. After incubation of the cells with each biomaterial, the supernatants were discarded and cells were washed with PBS, incubated with the Live/Dead solution for 15 min as indicated by the manufacturer, and washed with PBS. Samples were then observed by fluorescence microscope (Nikon Eclipse 90i, Nikon, Japan). For each experimental condition, five images were taken. The images were processed with *Image J* software (MacBiophotonics, Ontario, Canada) developed at McMaster University, in order to quantify the number of red (dead) and green (live) cells. For each condition, five independent determinations were taken and an average of 1,000 cells was analyzed.

Normalized average and standard deviation results were calculated by considering the results obtained for the positive controls as 0% mortality (100% survival) and the those attained for the negative controls as 100% mortality (0% survival).

The Kruskal-Wallis and the Mann-Whitney tests were used. Statistical significance was set at p<0.05.

## Results

### Zinc and calcium complexation

**Fig 1:**
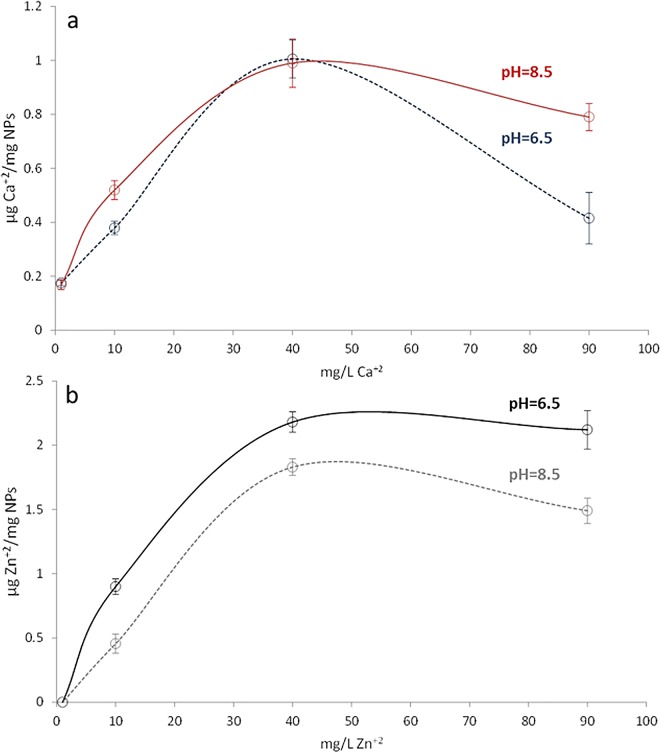
Zinc and calcium chelation ability of NPs. **a.** μg Ca^2+^/mg of NPs and **b.** μg Zn^2+^/ mg of NPs. Values were determined at two different pH values, measured by means of an inductively coupled plasma optical emission spectrometer. All tests were performed in triplicate. After Kruskal-Wallis and Mann-Whitney tests, all groups were significantly different (p<0.05), except when NPs were immersed in CaCl_2_ aqueous solutions containing 40 ppm at 6.5 or 8.5 pH values (p>0.05).

Calcium and zinc chelation ability of NPs is presented in Fig 1A and 1B. Significant differences exist in calcium quelation ability of NPs when the different tested concentrations were used (p<0.05), regardless of the employed pH; except when NPs were immersed in a CaCl_2_ aqueous solutions containing 40 ppm, at this point the pH value did not significantly changed the calcium quelation ability of NPs (P>0.05). When NPs were immersed in ZnCl_2_, significant differences were found for different concentrations and pH values (p<0.05). The maximum chelation capacities were obtained for NPs immersed in ZnCl_2_ or CaCl_2_ aqueous solutions containing 40 ppm of calcium or zinc at 6.5 pH value.

### Transmission electron microscopy (TEM) characterization

**Fig 2:**
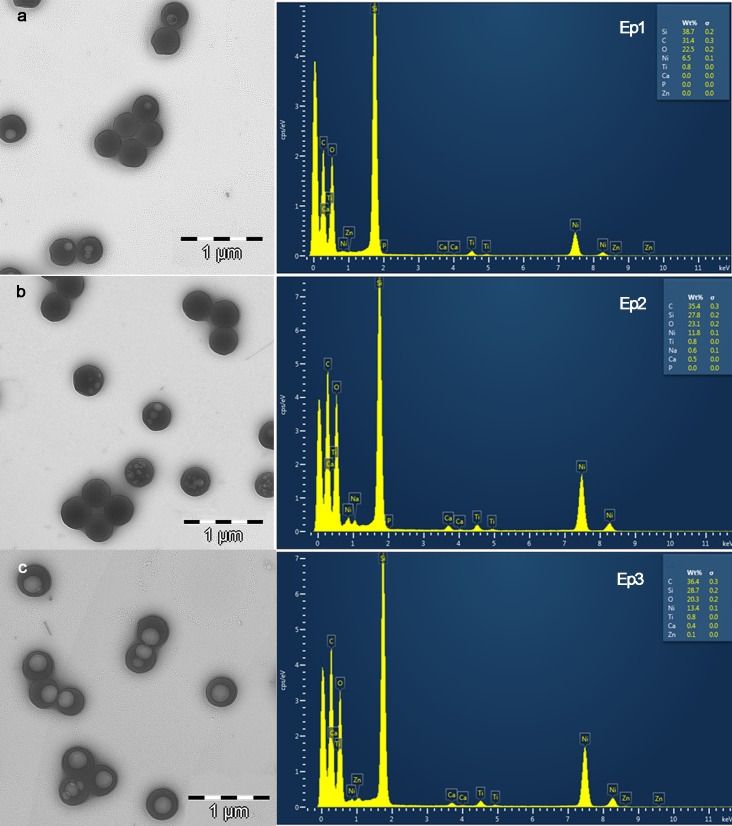
TEM images of NPs. **a.** Non-loaded NPs, zinc and calcium are absent in the EDX spectrum (Ep1). **b.** Calcium loaded NPs. Calcium is detected in the EDX spectrum analysis (Ep2) **c.** Zn loaded NPs. Zinc and calcium were detected in the EDX spectrum analysis (Ep3). Light circular objects inside of the NPs were artifacts that developed during electron beam transmission. Ni, Si and Ti in the EDX spectra were contaminant elements from the sample holder. Presented EDX spectra correspond to qualitative analysis and to determine the identification of the elements present and is not a normalized quantitative analysis.

Images and analytical spectra are displayed in Fig 2. Nps presented a spherical shape. No differences in morphology were found after zinc and/or calcium quelation. Elemental analysis on TEM confirmed the effective zinc and calcium quelation on NPs. Calcium was also identified in NPs that were zinc-loaded (Fig 2, Ep3).

### Acellular static in vitro bioactivity test

**Fig 3:**
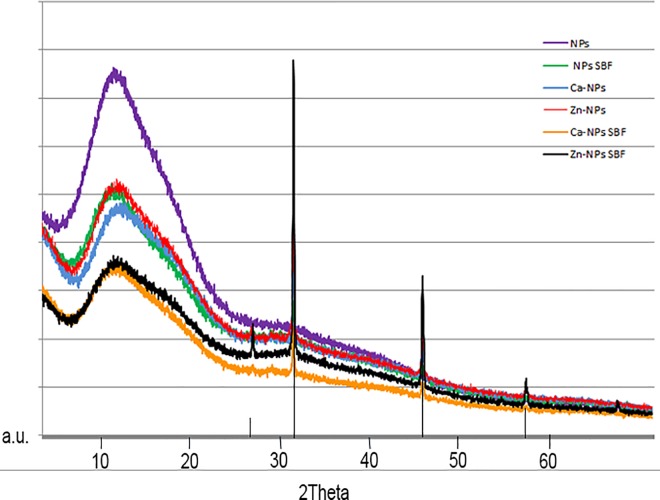
XRD spectra of NPs, Zn-NPs or Ca-NPs before and after 7 days of SBFS immersion. An amorphous crystallization pattern of calcium phosphate deposits is shown. Sodium chloride crystals formation (vertical bars) was also detected.

**Fig 4:**
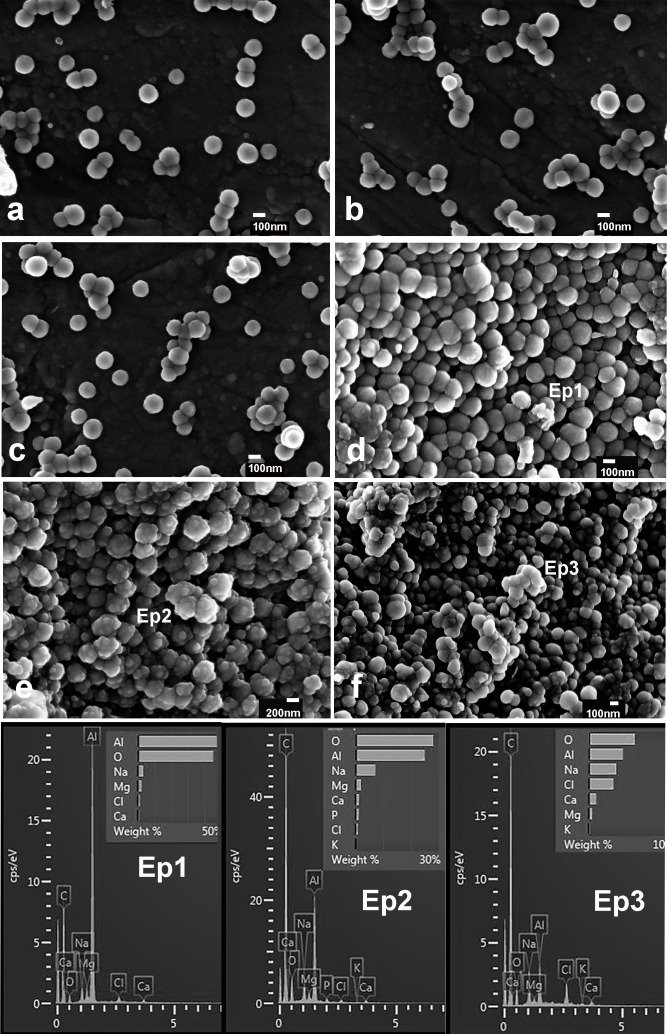
FESEM micrographs of NPs. **a.** Non-loaded NPs, before immersion in SBFS were spherical in shape. **b.** Ca-NPs, before immersion in SBFS. **c.** Zn-NPs, before immersion in SBFS. **d.** NPs after immersion in SBFS for 7 d. A negligible amount of calcium was encountered in EDX spectra (Ep1). **e.** Ca-NPs after immersion in SBFS for 7 d. Spotty calcium deposits were uniformly distributed throughout particles surfaces. Particles lost their regular morphology. Calcium and phosphorous were identified after EDX analysis (Ep2). **f**. Zn-NPs after 7 days immersion in SBFS. Calcium was identified after elemental analysis (Ep3). At the EDX spectra, chloride and sodium were detected after SBFS immersion; aluminum and magnesium were contaminant elements from the sample holder. Presented EDX spectra correspond to qualitative analysis and to determine the identification of the elements present and is not a normalized quantitative analysis.

XRD patterns of NPs, Ca-NPs and Zn-NPs before and after SBFS immersion are presented in Fig 3. Amorphous composition of calcium and phosphate deposits was evidenced. Some crystal formations were encountered in spectra, corresponding to sodium chloride. FESEM images are presented in Fig 4. Particles were spherical in shape (Fig 4A). Calcium and phosphorous deposits were detected, by EDX, only on Ca-NPs (Fig 4C). These deposits were encountered as rounded agglomerates onto the NPs surfaces. After surface calcium phosphate depositions occurred, NPs lost their round and homogeneous shape (Fig 4C). No traces of zinc could be evidenced by the EDX analysis performed through the FESEM microscope.

### Cell viability analysis

**Fig 5:**
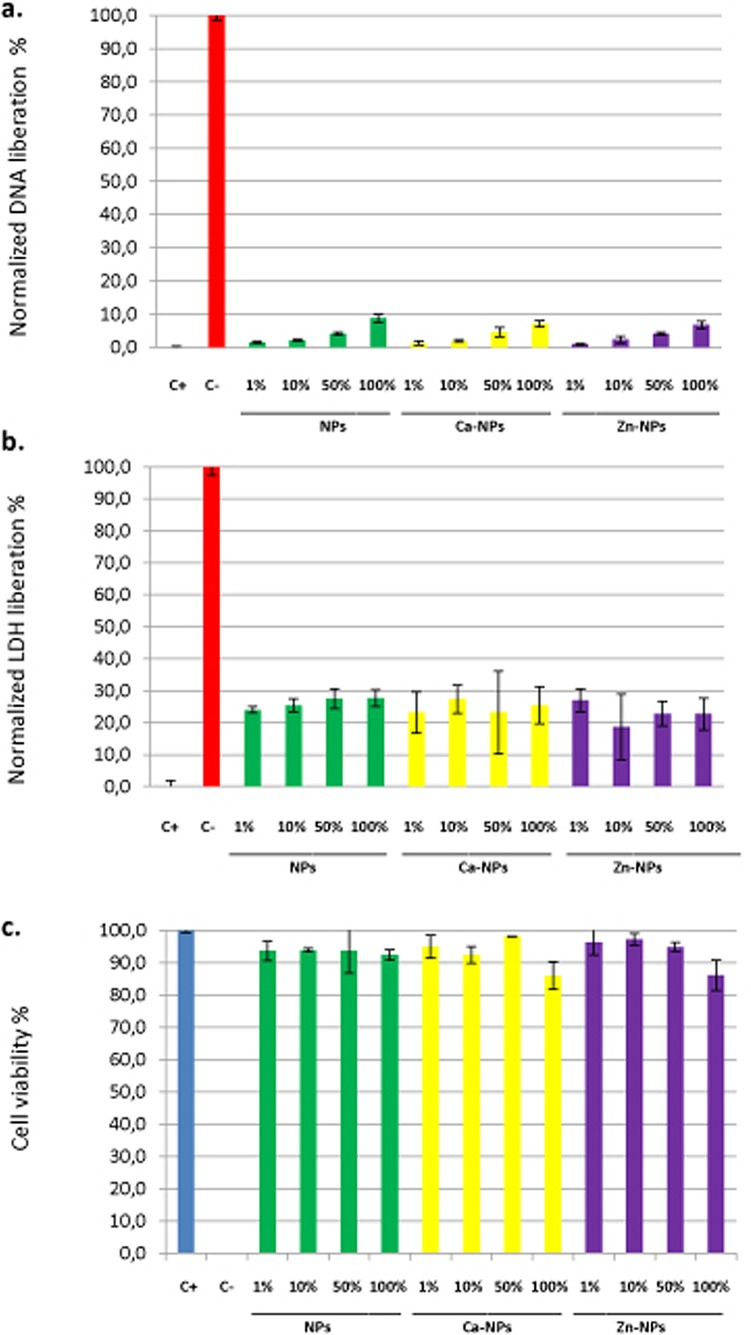
**a. Normalized average percentage of DNA liberation of human fibroblasts to the culture medium for the different experimental groups.** Mean values and standard deviations of 3 independent experiments for each experimental group are presented. C+ and C–are positive and negative control for cytotoxicity respectively. All the experimental groups were significantly different from controls (p<0.05). The rest of the groups were equal except for NPs 1% group that was different from Zn-NPs 1% (p<0.05), and NPs 100% group that was different from Zn-NPs 100% (p<0.05), being Zn-NPs less toxic to cells. **b. Normalized average percentage of LDH liberation of human fibroblasts to the culture medium for the different experimental groups.** Mean values and standard deviations of 5 independent experiments for each experimental group are presented. C+ and C–are positive and negative control for cytotoxicity respectively. All the experimental groups were significantly different from controls (p<0.05). The rest of the groups were similar (p>0.1). **c. Average cell viability according to the Live/Dead assay in human fibroblasts cells incubated with different NPs dilutions and controls.** Mean values and standard deviations of 5 independent experiments for each experimental group are presented. C+ and C–are positive and negative control for cytotoxicity respectively. All the experimental groups were significantly different from controls (p<0.05). The rest of the groups were equal except for NPs 100% group that was less cytotoxic than Zn-NPs 100% and Ca-NPs 100% groups (p<0.01). 1, 10, 50 and 100% dilutions correspond to 30, 15, 3 and 0.3 mg of NPs/ml respectively. Ca-NPs contain 0.99 μg of Ca^2+^/mg of NP and Zn-NPs contain 2.18 μg of Zn^2+^/mg of NP.

**Fig 6:**
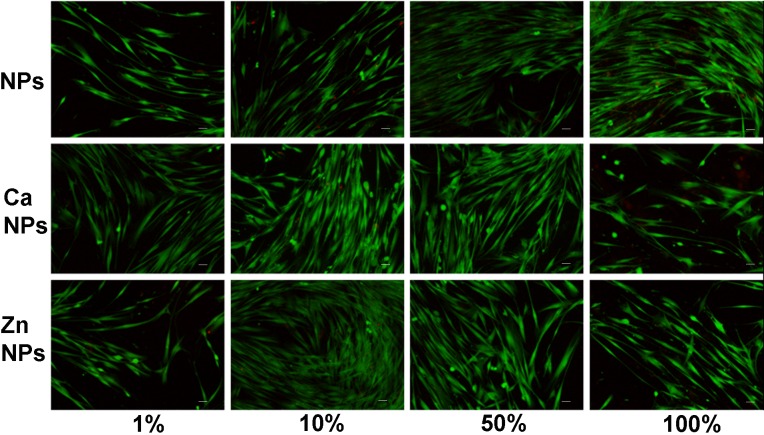
Fluorescence microscopy images corresponding to the analysis of cell viability according to the LIVE/DEAD assay, in human fibroblast cells, incubated with different NPs dilution for 24 h. Green cells correspond to live cells whereas dead cells are stained in red. **a, b, c** and **d** are cells exposed to 1,10,50 and 100% NPs dilutions. **e**, **f, g** and **h** correspond to cells exposed to calcium loaded NPs at 1, 10, 50 and 100% dilutions. **i, j, k,** and **l** are cells incubated with 1, 10, 50, and 100% dilutions of zinc loaded NPs. 1, 10, 50 and 100% dilutions correspond to 30, 15, 3 and 0.3 mg of NPs/ml respectively. Ca-NPs contain 0.99 μg of Ca^2+^/mg of NP and Zn-NPs contain 2.18 μg of Zn^2+^/mg of NP.

Results from cytotoxicity assays are shown in Fig 5. Three different techniques were used: 1) quantifying the liberated deoxyribonucleic acid from dead cells, 2) detecting the amount of lactate dehydrogenase enzyme released by cells with damaged membranes, and 3) by examining the cytoplasmic esterase function and cell membranes integrity with a fluorescence-based method using the Live/Dead commercial kit; and for the three different techniques, all the experimental groups were significantly different from both controls (p<0.05). Liberated DNA to the culture media was found to be under 10% -if normalized with the control groups- for all types of NPs and dilutions. A dose-dependent tendency was noticed in liberated DNA. The different NPs attained similar values, except for NPs 1% group that was different from Zn-NPs 1% (p<0.05), and NPs 100% that was different from Zn-NPs 100% (p<0.05), being in both cases Zn-loaded NPs less toxic to cells (Fig 5A). According to LDH enzyme quantification in the culture medium, normalized values were always under 30%, and were similar independently of the NPs type or dilution (p>0.1) (Fig 5B). And finally, after examining the integrity of the cytoplasmic esterase function and cell membrane integrity with a fluorescence-based method (Live/Dead), human fibroblasts viability was always above 90%, except for Ca-NPs and Zn-NPs at the highest concentration (100% -30 mg/ml-) that attained cell viability around 86% (p<0.01)(Fig 5C). Fluorescence microscopy images for each experimental group are presented in Fig 6. After analysis of cytotoxic cell damage induced by the three experimental NPs may be stated that NPs produced a negligible degree of cytotoxicity (Figs 5 and 6). The average LD50 (lethal dose 50%) was above 30 mg/ml for all tested NPs.

## Discussion

The null hypotheses have to be rejected, as calcium and zinc quelation on polymeric NPs are possible, in a sustained and pH dependent manner. Biomimetic precipitation of calcium phosphate deposits is produced on calcium-loaded polymeric spheres after immersion in SBFS and NPs permit human fibroblasts viability.

Calcium and zinc-loading on polymeric nanoparticles was effective and did not modify NPs morphology (Figs 1 and 2). The surface of these particles contained functional groups (COO^-^) able to complex metal cations. Particles immersed in ZnCl_2_ solution acquired zinc and calcium (Fig 2C), due to the potent chelating effect of carboxyl groups [26], and to the existence of calcium impurities into the ZnCl_2_ solution, as described in detail previously [21]. However, tested nanoparticles have higher chemical affinity for zinc than for calcium (Fig 1). A possible explanation may lie in the different stability of the complexes formed by zinc and calcium with the carboxylate groups of NPs. In our systems, the formation constant of (COO)_2_Zn in the NPs surface, is higher than the formation constant of (COO)_2_Ca, and this fact could be due to the different physical properties of each ion; the ionic radii of calcium and Zn are 0.099 nm and 0.074 nm respectively. The differences between the ionic radii involve a different charge/radius ratio, and thus a different ligand affinity.

A material is considered bioactive if it is able to create bone-bonding with host bone tissue. Bioactivity can experimentally be predicted if Ca/P deposits are formed on the material surface, after SBFS immersion [22]. Calcium loading on NPs promoted biomimetic precipitation of Ca/P deposits during SBFS immersion. The process of Ca/P deposition may be explained, as the external surface of NPs contains carboxyl groups, which are negatively charged (potential zeta -41 ± 5 mV). After CaCl_2_ immersion, calcium complexation occurred (Fig 1) and it is hypothesized that calcium at the NPs surfaces ionically bonded to PO_4_^3^ ions (in SBFS), creating Ca/P deposits (Fig 4E). Moreover, the phosphate groups at the surface have under-coordinated oxygens, which lead to reactive surfaces that will attract calcium ions from SBF solution [27,28]. Different concentrations of CaCl_2_ solutions have been previously employed to produce bioactive polymers [29,30]. The presented approach consists of using functional molecules that can selectively induce the nucleation of Ca/P compounds, by tuning the chemistry of the particle. Controlling the nucleation of a mineral phase will result in the synthesis of hybrid particles, composed of two different materials (*e*.*g*. polymers and inorganic compounds)[31].

Biomimetic remineralization of the tested NPs is crucial in periodontal regeneration. It has been previously shown that inorganic materials (HAp and other calcium phosphates), have been used for periodontal regeneration [4,17,18]. Calcium phosphate has excellent properties including: (1) a similar composition to bone minerals; (2) the ability to form bone apatite-like materials or carbonate HAp; (3) is able to stimulate cells, leading to the formation of bone; and (4) osteoconductivity [32]. During bone metabolism, osteoclasts release Ca^2+^ and PO_4_^2-^ derived from the mineralized matrix, causing a local increase in ion concentrations, in the microenvironment, which plays a role in osteoblast proliferation and differentiation. Increases in extracellular Ca^2+^ concentrations are potent chemical signals for cell migration and growth [33], and for bone remodeling [34]. PO_4_^2-^ is also a regulator of osteoblast proliferation and differentiation [35]. Periodontal ligament and pulp cells stimulated with extracellular Ca^2+^ and for 24 h, PO_4_^2-^ also increased bone morphogenetic protein-2 (BMP-2) mRNA expression [5,36].

To accomplish the restoration of the original architecture of the periodontal apparatus, it is important to promote not only osteogenesis but also cementogenesis, rapidly on the root surface after root planning/conditioning, because the cementum is the only hard tissue that can insert periodontal ligaments and assists in anchoring the tooth to the surrounding alveolar bone [5,16]. According to the anatomical location, which is in proximity to osteoblasts from alveolar bone, but is separated by the periodontal ligament, cementoblasts may be under a specific microenvironment resembling bone. Thus, these cells may also react with changes in the concentrations of extracellular inorganic ions. Whether cementoblasts could sense extracellular Ca^2+^ and PO_4_^2-^ ionic concentrations and altered cell functions such as cementogenesis and cytokine production still remains unclear [37]. Increases in extracellular Ca^2+^ produced an augmentation in fibroblast growth factor-2 (FGF-2) levels. FGF-2 promotes bone and cementum formation, and enhances cementoblasts and periodontal ligament cells mitosis, leading to periodontal tissue regeneration. Periodontal ligament fibers with correct functional orientation are also formed under FGF-2 stimulation [38].

When these NPs have been previously applied on etched dentin, they bond to collagen. Negatively charged polymeric NPs (negative zeta potential -41 mV-) had a high affinity to the positively charged demineralized dentin collagen [39] and facilitate further dentin remineralization [40]. Similar events are expected when these NPs get in contact to cementum; this issue deserves further research.

It is important to emphasize that encountered Ca/P deposits on polymeric NPs were amorphous in nature (Fig 3). Amorphous Ca/P provides an ion-rich environment that is favorable for *in situ* generation of prenucleation clusters, succeeding further remineralization [41]. On the contrary, if crystalline calcium phosphates are formed or incorporated into biomaterials, they will have non-controllable degradation times, requiring days, months or even years to provide ions to the media [31]. Usually, ceramics included in biomaterials are crystalline and release of Ca^2+^ and PO_4_^2-^ ions into their surroundings after implantation is impaired [42]. HAp has a lower dissolution rate at physiological pH (7.2–7.6) than other types of calcium phosphate, and this low dissolution behavior has been associated with lower osteoinductivity, resulting in poor biological response [42]. Other amorphous matrixes, as nano/micro-sized calcium phosphate or beta-tricalcium phosphate, have also been tested but dissolution behaviors are not always as long-lasting as required, and degradation products may not be completely cytocompatible [5,6].

NPs are expected to fill a specific anatomic defect to allow the further localization of cells, to serve as scaffolding for the formation of new tissue. Therefore, analyzing cell-NPs interactions is compulsory as many cellular and molecular events are involved in periodontal tissue repair/regeneration. These nanoparticles are composed by 2-hydroxyethyl methacrylate, ethylene glycol dimethacrylate and methacrylic acid, connected covalently. Altogether, the synthesis is characterized by a simple but efficient procedure and it is crucial the absence of harmful solvents, or non-polymerized compounds which are likely to later interfere with cellular biological processes. Even when these chemical compounds are expected to be cytocompatible, testing the effects of NPs on the viability of cells in culture has to be used as a predictor of potential toxic effects in whole animals. Previous studies suggested that material size and surface area play important roles in the observed cytotoxicity [43], and these effects are generated in the cells, but not by the ions found in the medium. Nanoparticles may, then, result in more cytotoxicity compared with larger ones (size effect) [43].

If cell viability analysis were considered altogether, data revealed that a correct identification of necrotic and apoptotic effects, of the different NPs, are possible combining different experimental techniques. The interpretation difficulties may very well reflect and highlight the importance of using a testing battery approach in cytotoxicity research [44]. For the interpretation of our findings, it should be taken into account that one single cell line was used, and it may not necessarily be extrapolated to other cell types [45].

With the exception of NPs, which were found to be non-toxic and non-apoptotic in all the assays, calcium and zinc-loaded particles presented a potentially, dose dependent but very low cytotoxic effect (Fig 5). Polymethylmethacrylate NPs have been previously shown to be non-cytotoxic [8]. Taken this and our results into account, and assuming that dissolution of these particles is unlikely, and unpolymerized monomers are absent, all toxicity observed will not be related to the NPs as such, and will be due to calcium or zinc ions on NPs. Calcium and zinc-loading polymers for periodontal regeneration have been intended before. Previously displayed results stood that calcium-loading of polymeric membranes (less than 15 wt. % of CaO NPs, relative to the total polymer weight) was not harmful to cells [14]. In addition, zinc oxide NPs inclusion on same polymers (5, 15, and 30 wt % of ZnO NPs, relative to the polymer weight) increased membranes cell cytotoxicity in a dose-dependent manner. Approximately 50% reduction in cells viability was obtained [13]. Even when direct comparisons are difficult to establish, due to different NPs chemistry and concentrations, our results only partially corroborate these findings, as we did find higher degree of cytocompatibility. In general, tested NPs regardless of zinc or calcium loading were shown to be non-cytotoxic and non-apoptotic in human fibroblasts in any assay, with the exception of the Live/Dead test in which 100% (30 mg/ml) Ca-NPs and Zn-NPs concentrations were shown to exert a slightly higher (10% increase) cytotoxicity respect to the other groups. But, cells viability was always above 80% (Figs 5 and 6). Only a small, but significant cytotoxic effect could be observed in the Live/Dead test. Whether, this is due to a particularly high sensitivity of this assay remains to be investigated. The cytotoxicity of ZnO NPs previously tested on human periodontal ligament fibroblast was found to strongly depend on concentration, being harmful at concentrations of 50 and 100 micrograms/mL [46]. It may be concluded that ionic zinc or calcium incorporation in the nanopolymer structure did exert lower cytotoxicity than when CaO or ZnO NPs are directly incorporated into the biomaterial [13,14].

However, it should be stressed that the required doses of Ca- and Zn-NPs to induce an *in vivo* effect remains to be ascertained. Even when high doses (up to 30 mg/ml) have been evaluated, this issue is essential to understand if the assessed dose ranges tested in this *in vitro* assay have physiological significance.

It should be also considered, that calcium-loaded NPs in SBFS will have their surfaces covered by calcium phosphate. It is expected that calcium and phosphate covered NPs will be even less harmful to cells. A low inherent toxicity of other calcium phosphate nanoparticles, have been previously reported [47] and have been proposed for periodontal regeneration [48,49]. Modification of injectable poly(methylmethacrylate)-based bone cement with nano-HAp incorporation did also show an enhanced degree of cells biocompatibility [50]. Moreover, it has been previously published that small particle sized, spherical and covered with calcium-phosphate is the best surface coating to use in experiments to quickly expand the cell population and maintain cell phenotype [51]. Recent works indicate that composite scaffolds made of ceramics/polymers are undoubtedly more effective than the single counterparts in terms of osteoconductivity, osteogenicity and osteoinductivity [52,53]. Therefore, the nanoparticles used in this study represent a promising tool for therapeutic approaches in periodontal regeneration.

Tested NPs have been previously shown to inhibit MMPs [21]. Antibacterial properties, and the possibility of incorporating proteins (*e*.*g*. mineralizing factors, enzymes inhibitors …) through binding or adsorption are possible, due to the surface chemistry of tested NPs, and it deserves future research. These issues will also provide crucial cues to periodontal tissue repair and bone regeneration.

## Supporting Information

S1 File Excel file with Ca and Zn complexation data. (XLSX)

S2 File Excel file with Cell viability data. (XLSX)
